# A Rare Stapes Abnormality

**DOI:** 10.1155/2015/387642

**Published:** 2015-01-05

**Authors:** Hala Kanona, Jagdeep Singh Virk, Gaurav Kumar, Sanjiv Chawda, Sherif Khalil

**Affiliations:** ^1^ENT Department, Queen's Hospital, Barking, Havering and Redbridge University Hospitals NHS Trust, Rom Valley Way, Romford, Essex RM7 0AZ, UK; ^2^Radiology Department, Queen's Hospital, Essex RM7 0AG, UK

## Abstract

The aim of this study is to increase awareness of rare presentations, diagnostic difficulties alongside management of conductive hearing loss and ossicular abnormalities. We report the case of a 13-year-old female reporting progressive left-sided hearing loss and high resolution computed tomography was initially reported as normal. Exploratory tympanotomy revealed an absent stapedius tendon and lack of connection between the stapes superstructure and footplate. The footplate was fixed. Stapedotomy and stapes prosthesis insertion resulted in closure of the air-bone gap by 50 dB. A review of world literature was performed using MedLine. Middle ear ossicular discontinuity can result in significant conductive hearing loss. This can be managed effectively with surgery to help restore hearing. However, some patients may not be suitable or decline surgical intervention and can be managed safely conservatively.

## 1. Introduction

Middle ear malformations occur in approximately 1 in 10000 births and can lead to severe conductive hearing loss [1]. The commonest congenital ossicular abnormalities are thought to include stapes fixation and incudostapedial discontinuity [2]. Patients classically present with nonprogressive hearing loss, as opposed to progressive hearing loss, which is more in keeping with acquired disease.

A wide range of congenital ossicular abnormalities are described in the literature, including absence of the stapes, stapes suprastructure, stapedius tendon, incus, and oval window alongside fixation of the stapes to the promontory and fallopian canal, as well as various malformations of the malleus, incus, and stapes [3–9]. These abnormalities are usually described as ossicular discontinuity, ossicular fixation, or both. A classification system for congenital abnormalities within the middle ear was developed by Cremer and Teunissen (Table 1) [10]. Management may be nonsurgical or surgical. Reconstructive options are summarised in Table 2 [11].

We present an extremely rare variant of stapes abnormality that leads to severe conductive hearing loss.

## 2. Case Report

**Figure 1:**
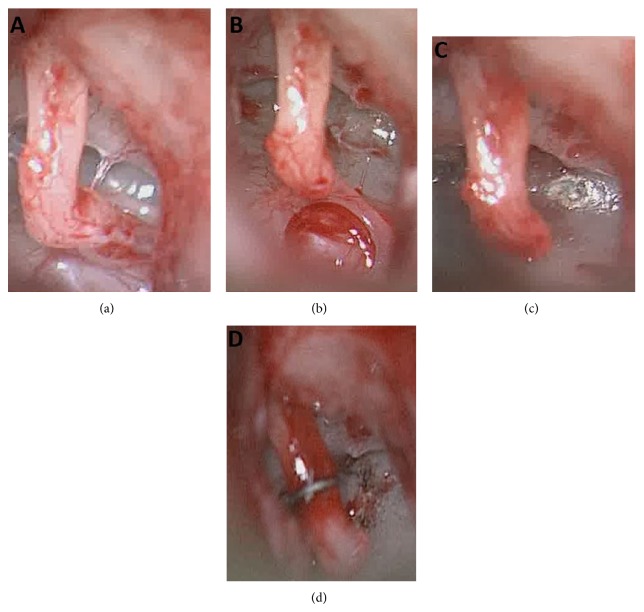
Intraoperative microscopic view of middle ear (a). Following tympanotomy, note lack of connection between stapes superstructure and footplate alongside absence of stapedius tendon (b). Following dislocation of incudostapedial joint (c). Following laser stapedotomy (d). Following placement of prosthesis and crimping by KTP laser.

**Figure 2:**
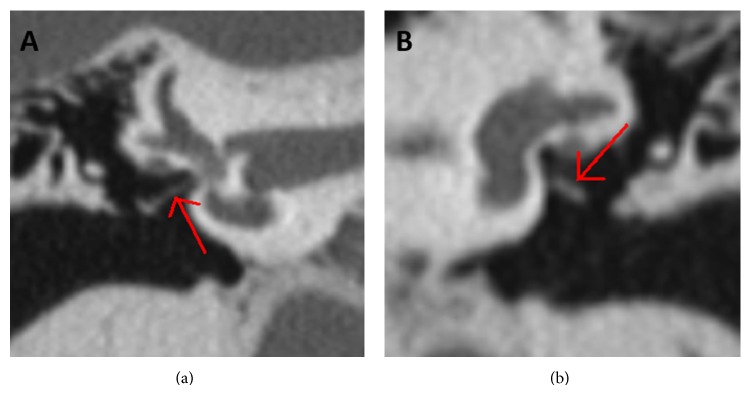
(a) Coronal section of CT showing normal position of stapes and tympanic portion of facial nerve on the right ear. (b) Coronal section of CT showing dislocation of stapes suprastructure from footplate on the left ear.

A 13-year-old female presented with a 2-year history of progressive left-sided hearing loss. There were no associated otological symptoms or history of infection or trauma. The ear drum was intact and normal. Pure tone audiometry elucidated a maximal air-bone gap and conductive hearing loss with a 4-frequency (0.5, 1, 2, and 4 kHz) mean of 68 dB on the left with normal thresholds on the right. High resolution computed tomography (HRCT) of the temporal bones was initially reported as normal. The patient however elected for exploratory tympanotomy and this demonstrated lack of connection between the stapes suprastructure and the footplate, which was fixed, alongside an absent stapedius tendon (Figure 1). A stapedotomy was performed and prosthesis then inserted (*SMart piston*, Olympus, Southend, UK). Further review of preoperative imaging indicated this ossicular abnormality (Figure 2). Postoperative follow up at 3 months confirmed closure of the air-bone gap by 50 dB with 4-frequency mean air conduction of 26 dB.

## 3. Discussion

This case demonstrates an extremely rare congenital ear abnormality. Interestingly, this patient presented atypically with progressive rather than nonprogressive conductive hearing loss. Disconnection of the stapes superstructure from the footplate has only been reported once in the literature [12]. Atresia of the oval window has been more commonly documented [13].

Congenital middle ear abnormalities can occur independently but often occur in patients with anomalies of the external ear or with craniofacial dysplasia [1]. Syndromes affecting development of the first and second branchial arches can affect the auricle, external ear canal, and ossicular chain. Hypoplasia and malformation of the middle ear are associated with Branchio-oto-renal syndrome and Crouzon's syndrome [14]. Isolated inherited ossicular abnormalities have also been reported, such as complete absence of the long process of the incus and fixation of the stapes alongside congenital absence of the stapes and oval window in two siblings [15, 16]. The genetics remain poorly understood however.

Within the literature, there are differing schools of thought concerning the embryology of the middle ear. It is widely accepted that the ossicles arise from the mesoderm of the first and second branchial arches (Meckel's and Reichert's cartilage, resp.). However, there is conflicting literature regarding the exact embryological development [17–19]. The first branchial arch gives rise to the head of malleus and short process and body of incus. The second branchial arch gives rise to the lateral process of the malleus, long process of the incus, and the stapes suprastructure. The stapes footplate originates from the otic capsule, which, in turn, arises from the neurectoderm [17–19]. One study examining 20 embryos showed that the stapedial “anlage” (cluster of embryonic cells) develops independently from both Reichert's cartilage and the otic capsule. Instead, the stapedial anlage is separated from Reichert's cartilage by an interhyale, a segment that gives rise to the tendon of the stapedius muscle [18]. The superior aspect of the anlage then gives rise to the stapes footplate, and the inferior aspect to the stapes suprastructure [18]. When one considers disconnection of the stapes superstructure from the footplate (as described in our case), it seems perhaps more plausible to agree with the former embryological description, rather than the latter embryological study.

Patients with ossicular abnormalities characteristically display a conductive hearing loss. This can be up to 60 dB or maximal, especially in those with oval window atresia [19]. The gold standard investigation remains HRCT and can accurately diagnose oval window atresia, for example [2, 20]. However, it is important that these images are reviewed meticulously by experienced clinicians and radiologists as negative reports, as evidenced by our case, do not exclude subtle malformations. In addition, current literature is now focusing on the potential role of cone beam CT, which has demonstrated similar efficacies with a lower radiation dose as compared with HRCT [21]. Malposition of the facial nerve is often associated with oval window atresia [20, 22]. It has been suggested that this may prevent contact between the stapes and otic capsule, thus inhibiting normal development of both structures [23].

The management of congenital ossicular abnormalities is naturally dependent upon the ossicular malformations present alongside technical and patient factors. Nonsurgical intervention should always be considered. It is generally accepted that in patients with oval window atresia, stapedotomy is the best surgical option (Table 2) [24]. Exploratory tympanotomy therefore remains a valid option even in the presence of “normal” or negative imaging.

## 4. Summary

- Congenital ossicular abnormalities are rare causes of conductive hearing loss in childhood and are important in the differential diagnoses for children presenting with nonprogressive hearing loss.
- Congenital ossicular malformations can often occur in patients with syndromes and a relatively common malformation includes oval window atresia.
- High resolution computed tomography is the imaging modality of choice and should be reviewed meticulously by experienced clinicians and radiologists.
- Management options are either conservative or surgical with exploratory tympanotomy, with or without ossicular reconstruction.

## Figures and Tables

**Table 1:** Classification of congenital ossicular malformations (adapted from Teunissen and Cremers [10]).

Class I	Isolated stapes footplate fixation
Class II	Stapes fixation in combination with a congenital anomaly of the ossicular chain
Class III	Anomalies of the ossicular chain and mobile stapes footplate
Class IV	With aplasia or severe dysplasia of the oval window or round window

**Table 2:** Reconstructive options for ossicular chain discontinuity (adapted from Bhatti and Bluestone [11], surgical atlas of paediatric otolaryngology pages 75–77).

Absent ossicle(s)	Recommended reconstructive options
Malleus	Autograft incus
	Type II tympanoplasty

Incus	Autograft cartilage
	Incus prosthesis
	Type III tympanoplasty

Stapes superstructure	Autograft incus
	Incus-stapes prosthesis

Malleus and incus	Autograft cartilage
	Type III tympanoplasty
	PORP

Incus and stapes superstructure	Autograft cartilage
	Incus-stapes prosthesis

Malleus, incus, and stapes superstructure	Autograft cartilage
	TORP

PORP = partial ossicular replacement prosthesis; TORP = total ossicular replacement prosthesis.
